# The Relationship Between Maternal and Neonatal Microbiota in Spontaneous Preterm Birth: A Pilot Study

**DOI:** 10.3389/fped.2022.909962

**Published:** 2022-07-22

**Authors:** Chiara Tirone, Angela Paladini, Flavio De Maio, Chiara Tersigni, Silvia D’Ippolito, Nicoletta Di Simone, Francesca Romana Monzo, Giulia Santarelli, Delia Mercedes Bianco, Milena Tana, Alessandra Lio, Nicoletta Menzella, Brunella Posteraro, Maurizio Sanguinetti, Antonio Lanzone, Giovanni Scambia, Giovanni Vento

**Affiliations:** ^1^Fondazione Policlinico Universitario A. Gemelli IRCCS, U.O.C. di Neonatologia, Dipartimento di Scienze della Salute della Donna, del Bambino e di Sanità Pubblica, Rome, Italy; ^2^Istituto di Clinica Pediatrica, Università Cattolica del Sacro Cuore, Rome, Italy; ^3^Dipartimento di Scienze di Laboratorio e Infettivologiche, Fondazione Policlinico Universitario A. Gemelli IRCCS, Rome, Italy; ^4^Dipartimento di Scienze Biotecnologiche di Base, Cliniche Intensivologiche e Perioperatorie, Università Cattolica del Sacro Cuore, Rome, Italy; ^5^Fondazione Policlinico Universitario A. Gemelli IRCCS, U.O.C. di Ostetricia e Patologia Ostetrica, Dipartimento di Scienze della Salute della Donna, del Bambino e di Sanità Pubblica, Rome, Italy; ^6^Università Cattolica del Sacro Cuore, Istituto di Clinica Ostetrica e Ginecologica, Rome, Italy; ^7^Department of Biomedical Sciences, Humanitas University, Milan, Italy; ^8^IRCCS Humanitas Research Hospital, Milan, Italy; ^9^Dipartimento di Scienze della Vita e Sanitá Pubblica, Universitȧ Cattolica del Sacro Cuore, Rome, Italy

**Keywords:** microbiota, vagina, meconium, lung, preterm birth (PTB), bronchopulmonary dysplasia (BPD), bronchoalveolar lavage fluid (BALF)

## Abstract

The newborn’s microbiota composition at birth seems to be influenced by maternal microbiota. Maternal vaginal microbiota can be a determining factor of spontaneous Preterm Birth (SP^PTB^), the leading cause of perinatal mortality. The aim of the study is to investigate the likelihood of a causal relationship between the maternal vaginal microbiota composition and neonatal lung and intestinal microbiota profile at birth, in cases of SP^PTB^. The association between the lung and/or meconium microbiota with the subsequent development of bronchopulmonary dysplasia (BPD) was also investigated. Maternal vaginal swabs, newborns’ bronchoalveolar lavage fluid (BALF) (1st, 3rd, 7th day of life) and first meconium samples were collected from 20 women and 23 preterm newborns with gestational age ≤ 30 weeks (12 = SP^PTB^; 11 = Medically Indicated Preterm Birth–MI^PTB^). All the samples were analyzed for culture examination and for microbiota profiling using metagenomic analysis based on the Next Generation Sequencing (NGS) technique of the bacterial 16S rRNA gene amplicons. No significant differences in alpha e beta diversity were found between the neonatal BALF samples of SP^PTB^ group and the MI^PTB^ group. The vaginal microbiota of mothers with SP^PTB^ showed a significant difference in alpha diversity with a decrease in Lactobacillus and an increase in Proteobacteria abundance. No association was found between BALF and meconium microbiota with the development of BPD. Vaginal colonization by Ureaplasma bacteria was associated with increased risk of both SP^PTB^ and newborns’ BPD occurrence. In conclusion, an increase in α-diversity values and a consequent fall in Lactobacillus in vaginal environment could be associated to a higher risk of SP^PTB^. We could identify neither a specific neonatal lung or meconium microbiota profiles in preterm infants born by SP^PTB^ nor a microbiota at birth suggestive of subsequent BPD development. Although a strict match has not been revealed between microbiota of SP^PTB^ mother-infant couples, a relationship cannot be excluded. To figure out the reciprocal influence of the maternal-neonatal microbiota and its potential role in the pathogenesis of SP^PTB^ and BPD further research is needed.

## Introduction

Spontaneous preterm birth (SP^PTB^) represents the first cause of perinatal mortality and a huge public health cost in many countries, including Italy.

SP^PTB^ is a complex multifactorial obstetric and neonatological complication, and little is known about its heterogeneous pathogenesis (1). Although a proven infection can only be documented in about 40% of preterm births with premature membrane rupture or spontaneous labor, the colonization of the genital tract by some pathogens and chorioamnionitis have been traditionally considered as the most prevalent causes of SP^PTB^.

Accumulating evidence suggests that the uterine environment is not sterile but populated by a set of different microbial communities present in the placenta, in the fetal membranes and in the amniotic fluid that could contribute to the formation of the neonatal microbiota before birth (2–4).

The formation of the microbiota begins early at the time of organogenesis and can promote protection or predisposition toward certain insults and pathologies (5–7).

Nevertheless, it is known that fetal and maternal colonization process can be influenced by several factors such as the mode of delivery [vaginal delivery (VD) vs. cesarean section (CS)] (8, 9), maternal bonding, admission to intensive care unit, administration of antibiotic therapy, H_2_ antagonists, dietary modifications with formulated milk, use of fortifying breast milk and fasting periods (10, 11). These are frequent circumstances in premature infants, who often undergo treatments that can alter the eubiotic colonization process.

The evolution of infants’ gut microbiota was a very investigated topic in recent years, while few researchers have addressed the development of the microbiota of the respiratory tract, particularly lower airways one. Pulmonary colonization starts *in utero* and can be subsequently influenced by the exposition to the birth canal and the skin of the mother, as well as by microorganisms, introduced through ventilation or micro-aspiration (2, 8, 12–14). The lung microbiota shows changes in the first week of life (15, 16) and an evolution of the colonization pattern is found over the first week of life with evidence of the prevalence of organisms of the species *Staphylococcus* sp. (Firmicutes) or *Ureaplasma* spp. (Tenericutes) within 7 days (17).

Furthermore, lung microbiota can be influenced by lung diseases, particularly by the development of bronchopulmonary dysplasia (BPD) (16).

The aim of the study is to investigate the role of maternal and neonatal microbiota in SP^PTB^ cases. We investigated maternal vaginal microbiota, and neonatal lung and intestinal microbiota. We compared preterm infants born by spontaneous birth for suspected chorioamnionitis and/or premature prolonged rupture of the membranes (p-PROM) and/or preterm labor (SP^PTB^ group) with preterm infants born by cesarean section performed for maternal or fetal medical indications, with intact membranes and in the absence of labor (Medically Indicated Preterm Birth–MI^PTB^).

The identification of a specific maternal and preterm newborns microbiota signature associated to SP^PTB^ and to adverse neonatal outcomes, could open to the investigation of new markers (a) in pregnancy, to select cases at higher risk of SP^PTB^ and (b) in preterm newborns, to early identify cases at higher risk of adverse pulmonary outcomes.

## Materials and Methods

### Setting

We conducted a prospective observational study at the Department of Woman and Child Health of the Fondazione Policlinico Universitario A. Gemelli IRCCS of Rome (Italy), from August 2019 to August 2020. The study protocol was approved by the Institutional Ethics Committee in July 2019 (study ID 2400, protocol number 0031183/19). Written parental consent was obtained prior to study entry.

### Study Population and Inclusion Criteria

The study population included women undergoing PTB and their preterm infants having gestational age (GA) ≤ 30 weeks, who were intubated at birth or in the first 24 h of life.

As this was a pilot study, sample size was not calculated. As in 2018, 58 newborns with a GA ≤ 30 weeks were admitted to the Neonatal Intensive Care Unit (NICU), of them 26 (45%) were born for suspected chorioamnionitis and/or p-PROM and/or preterm labor (SP^PTB^ group) and 32 (55%) by elective C-section performed for maternal or fetal medical indications with intact membranes in the absence of labor (MI^PTB^ group). 70% (18/26) of newborns in SP^PTB^ group and 57% (18/32) in MI^PTB^ group were intubated at birth or in the first 24 h of life; we estimated the possibility of studying up to 36 newborns over a period of about 12 months. The number of infants we intend to study is in line with the available literature’s evidence for a pilot study (18).

The study population was furtherly divided into two groups:

SP^PTB^ group: preterm infants for suspected chorioamnionitis and/or p-PROM and/or preterm labor; and MI^PTB^ group: preterm infants born by CS for maternal or fetal medical indications, with intact membranes and in the absence of labor. BPD was defined as O_2_-dependence at 36 weeks of post menstrual age (19).

### Timing and Technique of Samples Collection

A vaginal swab was collected at the time of delivery for all the enrolled women. Newborns’ bronchoalveolar lavage fluid (BALF) and meconium specimens were collected. BALF were obtained after ensuring that the infant was adequately oxygenated by instilling 1 ml/kg of 0.9% sodium chloride in the endotracheal tube and suctioning the fluid into a sterile mucus trap. The first BALF sample was collected within the first 24 h of life, whereas further collections were performed on the third and seventh day of life in newborns who were still intubated.

Vaginal swab, BALF and meconium were stored at −80^°^C until further processing.

### Data Collection

Data was extracted from electronic medical records. The cause of preterm birth was recorded to distinguish SP^PTB^ infants and MI^PTB^ ones.

Data collection included both prenatal and neonatal data. Among (a) prenatal data: results of previous cultural exams of vaginal swab and/or maternal urine culture; use of antibiotics during pregnancy; prescription of intrapartum antibiotics prophylaxis; prenatal steroids prophylaxis (defined as the administration of two doses of betamethasone within 24 h of each other, with the second dose administered at least 24 h but no more than 7 days before delivery), (b) neonatal data: gestational age at delivery, birth weight, incidence of neonatal BPD (defined as O_2_-dependence at 36 weeks of post menstrual age), characteristics of the neonatal respiratory pathology (number of the administered doses of surfactant, incidence of persistent pulmonary hypertension requiring treatment, incidence of successful extubation, defined as the lack of need for new tracheal intubation in the 72 h following extubation), duration of ventilatory support (hours of oxygen therapy, hours of mechanical ventilation, hours of non-invasive ventilation), time until initiation of antibiotic therapy, number of antibiotics cycles, time until initiation of oral feeding, incidence of intraventricular hemorrhage (IVH) grade > 2°, length of stay and survival, in addition to the perinatal infectious risk factors.

The incidence of blood stream infections, defined as a positive blood culture, and pneumonia, defined as the worsening respiratory symptoms (increase of FiO_2_ and/or of the ventilatory setting, increase of secretions from the endotracheal tube, persistent radiographic anomalies ≥ 24 h) with positive BALF culture, were also registered.

### Sequencing and Bioinformatic Analysis

For each sample (Vaginal swabs, BALF and meconium), bacterial DNA extraction was performed in a strictly controlled level-2 biological safety workplace using DANAGENE MICROBIOME DNA kits (Danagen-Bioted) according to manufacturer’s instructions (20). DNA was fluorometrically quantified (Qubit dsDNA high-sensitivity assay, Thermo Fisher Scientific), and then subjected to the 16S rRNA V3–V4 region amplification as described above (21, 22). The resulting amplicons were purified using Agencourt AMPure XP beads (Beckman Coulter) and indexed using the Nextera XT Index kit (Illumina). The indexed amplicons were equimolarly diluted and pooled. Sequencing was performed *via* the 2 × 300-bp paired-end protocol in the MiSeq instrument (Illumina).

Demultiplexed FastQ reads were analyzed using the QIIME2 (v.2020.6) microbiome analysis pipeline (23). Briefly, merged reads were trimmed before filtering and chimera removal to generate amplicon sequence variants (ASVs) using the DADA2 algorithm (24). Both the pre-fitted sklearn-based taxonomy classifier^1^ and SILVA 132 database^2^ were used for taxonomic ASVs’ annotation. Final data were pre-processed to remove mitochondrial sequences (25). We used R 4.0.2^3^ and phyloseq (26) statistical packages for downstream analyses of alpha (e.g., Shannon index) and beta (e.g., Jaccard distance) microbial community’s diversity. Before that, we normalized to median sum count each sample in order to restrict uneven sampling effects. Difference between groups according to alpha diversity metric was assessed using the Mann–Whitney *U*-test, whereas that according to Jaccard distance matrix-computed beta diversity metric was assessed using the permutational multivariate analysis of variance (PERMANOVA).

### Statistical Analysis

Statistical analysis was done using GraphPad PRISM Version 8.4.3 considering a value of *p* < 0.05 as statistically significant.

Categorical variables were compared using a two-tailed Fisher’s exact test, while the differences between the groups for continuous variables were tested by Mann-Whitney *U*-test for non-parametric data and the Student’s *t*-test for parametric data.

## Results

From August 2019 to August 2020, a total of 30 mothers and 35 newborns were enrolled at birth after receiving informed parental consent. Six mother-newborns couples were excluded because newborns were not intubated in the first 24 h of life. The study group consisted of 29 newborns, including 3 pairs of twins, and 26 mothers.

Twenty-nine BALF, 29 early meconium and 26 vaginal swabs samples were analyzed. Six BALF samples were excluded due to the low bacterial biomass. A total of 20 mothers (11 = SP^PTB^; 9 = MI^PTB^) and 23 neonates (12 = SP^PTB;^ 11 = MI^PTB^), were included in the final statistical analysis.

The demographics and clinical characteristics of infants included in the analysis are shown in Table 1. No significant differences were observed between the two groups, except for earlier initiation of antibiotic therapy in infants of the SP^PTB^ group [1 (1–2) vs. 2.5 (2–7) days of life, *p* = 0.01] and for the occurrence of premature prolonged rupture of the membranes (pPROM) which only registered in the SP^PTB^ group (*p* < 0.0001). In this group of neonates, only in two cases labor was activated without rupture of the membranes. A trend in terms of lower gestational age (27.0 ± 1.9 vs. 28.3 ± 1.1 weeks, *p* = 0.07) and of higher incidence of late-onset sepsis [6 (50%) vs. 1 (9%), *p* = 0.07] was found in the SP^PTB^ group compared to the MI^PTB^ group. All 23 infants survived to 36 weeks’ postmenstrual age (only 1 infant in MI^PTB^ died after 36 weeks postmenstrual age). Based on their pulmonary outcomes, the 23 infants were further classified as those who did not develop BPD (No BPD group, *n* = 15) or those who later developed BPD (BPD group, *n* = 8). The demographics and clinical characteristics of patients in these two categories are shown in Table 2, with significant differences in some characteristics and outcomes. In particular, infants in the BPD group had a lower GA and birth weight, had a greater number of episodes of blood stream infection and pneumonia during hospitalization and received more cycles of antibiotic therapy. In addition, infants in the BPD group needed oxygen therapy and non-invasive ventilation for a longer time than infants in the No BPD group, had a hemodynamically significant patent ductus arteriosus (PDA) more frequently, and their hospital stay was significantly longer.

**TABLE 1:** Demographic and clinical characteristics of the newborns studied.

	MI^PTB^ group (*N* = 11)	SP^PTB^ group (*N* = 12)	*P*
Gestational age (weeks)	28.3 ± 1.1	27.0 ± 1.9	0.07
Birth weight (g)	857 ± 229	940 ± 322	0.48
Male sex	6 (54)	7 (58)	>0.99
Pairs of twins	2 (18)	1 (8)	0.59
Vaginal delivery	0	3 (25)	0.22
PROM	0 (0)	10 (83)	**<0.0001**
Positive vaginal swab (of which GBS)	5 (0)	9 [1]	0.21
Antenatal corticosteroids	10 (91)	11 (91)	>0.99
Intrapartum antibiotic prophylaxis	3 (27)	6 (50)	0.40
Time until initiation of antibiotics (days)	2.5 (2–7)	1 (1–2)	**0.01**
Number of antibiotic cycles	2.3 ± 2.6	2.9 ± 2.1	0.52
Diagnosis of Late onset Sepsis	1 (9)	6 (50)	0.07
Diagnosis of pneumonia	3 (27)	5 (42)	0.67
Day of life of first pneumonia	1 (1–5)	3.5 (1–40)	0.42
Administered surfactant doses	1 (0–3)	1 (1–3)	0.88
Duration of mechanical ventilation (hours)	89 ± 206	270 ± 558	0.32
Duration of O_2_-therapy (hours)	326 ± 570	719 ± 941	0.24
Duration of non-invasive ventilation (hours)	833 ± 755	643 ± 728	0.54
Successful extubation	7 (64)	3 (25)	0.09
Persistent pulmonary hypertension	3 (27)	3 (25)	>0.99
Hemodynamically significant PDA	3 (27)	4 (33)	>0.99
Bronchopulmonary dysplasia (BPD)	3 (27)	5 (42)	0.67
Intraventricular hemorrhage (IVH > 2°)	1 (9)	5 (42)	0.15
Periventricular leukomalacia	0	3 (25)	0.22
Time until initiation of oral feeding (days)	5.4 ± 6.0	2.8 ± 1.1	0.18
Admission length (days)	75 ± 27	89 ± 49	0.41
Alive	10 (91)	12 (100)	0.48

*Values are expressed as mean ± SD and no. (%). p < 0.05 is statistically significant.*

**TABLE 2:** Demographics and clinical characteristics of patients with and without diagnosis of BPD.

	BPD group (*N* = 8)	No BPD group (*N* = 15)	*P*
Gestational age (weeks)	26.5 ± 1.5	28.2 ± 1.5	**0.02**
Birth weight (g)	715 ± 144	999 ± 286	**0.02**
M sex	6 (75)	7 (47)	0.38
Vaginal delivery	2 (25)	1 (7)	0.27
PROM	4 (50)	6 (40)	0.68
Positive vaginal swab (of which GBS)	6 (0)	8 (1)	0.40
Antenatal corticosteroids	8 (100)	13 (87)	0.53
Intrapartum antibiotic prophylaxis	3 (37)	6 (40)	>0.99
Time until initiation of antibiotics (days)	1.0 (1–7)	1.5 (1–7)	0.62
Number of antibiotic cycles	4 (1–9)	1 (0–4)	**0.05**
Diagnosis of Sepsis	5 (62)	2 (13)	**0.02**
Diagnosis of pneumonia	6 (75)	2 (13)	**0.006**
Day of life of first pneumonia	1 (3.5–40)	1 (1–1)	0.35
Administered surfactant doses	2 (1–3)	1 (0–3)	0.06
Duration of mechanical ventilation (hours)	481.6 ± 645.8	24.5 ± 47.4	0.08
Duration of O_2_-therapy (hours)	1335.0 ± 873.9	101.7 ± 206.2	**0.005**
Duration of non-invasive ventilation (hours)	1272.0 ± 647.4	446.8 ± 612.7	**0.01**
Successful extubation	1 (12)	9 (60)	0.07
Persistent pulmonary hypertension	4 (50)	2 (13)	0.13
Hemodynamically significant patent PDA	6 (75)	1 (7)	**0.002**
SP^PTB^	5 (62)	7 (47)	0.67
Intraventricular hemorrhage (IVH > 2°)	3 (37)	3 (20)	0.62
Periventricular leukomalacia	2 (25)	1 (7)	0.27
Time until initiation of oral feeding (days)	6.5 ± 6.5	2.8 ± 1.7	0.16
Admission length (days)	117 ± 47	64 ± 19	**0.01**
Alive	7 (87)	15 (100)	0.35

*Values are expressed as mean ± SD and no. (%). p < 0.05 is statistically significant.*

### Lung and Meconium Microbiota of Preterm Infants at Birth

A total of 38 BALF samples were collected: 23 were collected during the first 24 h of life, 10 on day 3 of life and 5 on day 7 of life.

**FIGURE 1:**
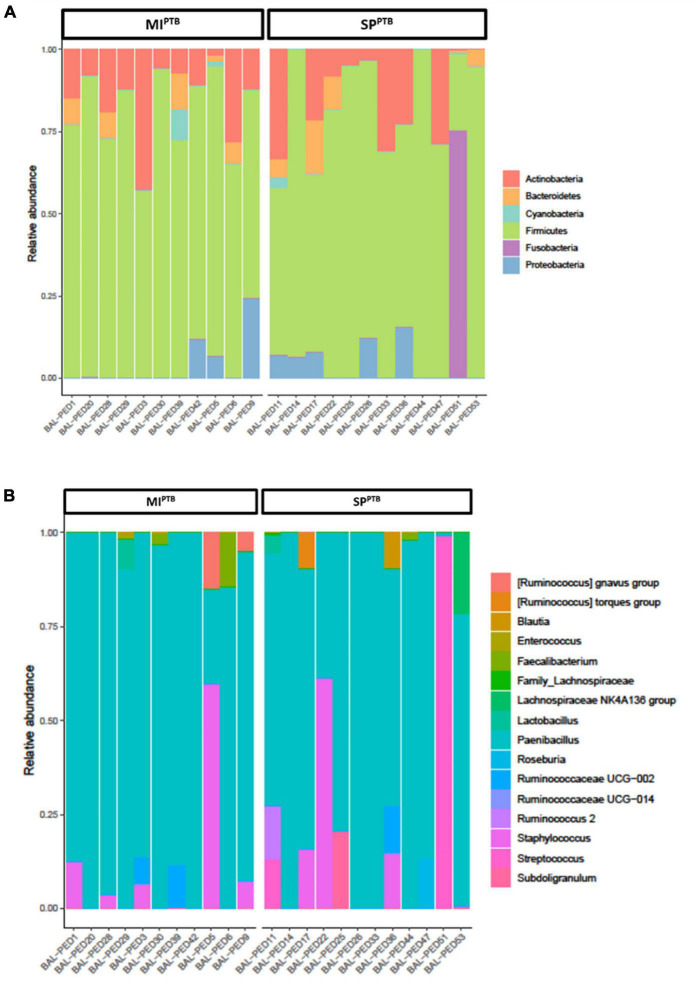
Relative abundances of bacterial taxa composing the lung bacterial communities in hospitalized newborns. For each sample of the MI^PTB^ and SP^PTB^ infants’ groups, proportions for major phyla **(A)** and major genera **(B)** were computed, normalized, and presented as stacked bar plots. The statistical significance at a *P*-value of ≤ 0.05 was assessed using the Mann–Whitney *U*-test.

We evaluated possible association between birth cause and lung microbiota composition investigating bacterial communities on both SP^PTB^ and MI^PTB^ groups in BALF specimens at day 1 post birth in intubated infants. A total of 1,146,747 high-quality sequences comprising 79 unique ASVs were obtained. All 38 sequenced samples showed yielded ≥ 1,000 sequences with a mean of 30,177 and median of 23,340, range 3,380–141,435 reads/sample. Overall, 6 main phyla were detected: *Actinobacteria, Bacteroidetes, Cyanobacteria, Fusobacteria, Firmicutes*, and *Proteobacteria*. In both groups, *Firmicutes* and *Actinobacteria* phyla prevailed and showed a comparable relative abundance average of 77 and 15% or 73 and 13% for SP^PTB^ and MI^PTB^, respectively. Conversely, *Proteobacteria* and *Bacteroidetes* were less represented, and *Fusobacteria* and *Cyanobacteria* were detected only in few specimens (Figure 1A). Similarly, no significant differences were observed at genus level where *Paenibacillus* or *Staphylococcus* genera were predominant (Figure 1B).

**FIGURE 2:**
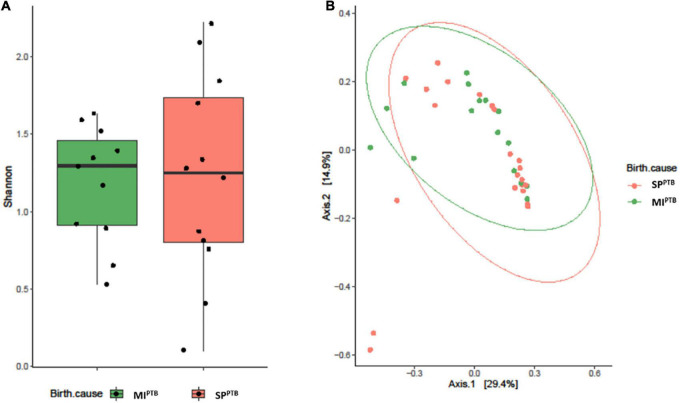
Alveolar microbiota Alpha and Beta diversity analysis of preterm newborns classified on the basis of preterm birth subtype (MIPTB and SPPTB). Alpha diversity was evaluated by Shannon index measure. The values obtained were compared, resulting in no statistically significant difference between MIPTB and SPPTB infants’ groups (Mann–Whitney *U*-test) **(A)**. Comparison for Beta diversity analysis was made using Jaccard distance. The principal coordinate analysis (PCoA) results are presented as two-dimensional ordination plots, which were generated using two principal coordinates (i.e., axis 1 and axis 2) **(B)**. The statistical significance at a *P*-value of ≤ 0.05 was assessed using the permutational multivariate analysis of variance (PERMANOVA).

Alpha diversity, measured by Shannon Diversity Index, and beta diversity, as Jaccard distance, were evaluated in SP^PTB^ and MI^PTB^ groups, as reported in Figures 2A,B. Shannon index showed comparable values in both groups: 1.85 ± 0.86 and 1.74 ± 0.42 for SP^PTB^ and MI^PTB^ (*p* > 0.05), respectively. Three couples of twins were included in the study: one in the SP^PTB^ and two in the MI^PTB^ group. Brothers had different values of alpha diversities: 1.22 and 1.69 for twins in the SP^PTB^ group; 1.35 and 0.89 and 1.29 and 0.92 for twins in the MI^PTB^ group. Turning to Beta diversity, these samples showed a different spatial distribution highlighting the importance of other factors in the establishment of a unique microbial composition. No difference in spatial distribution of the SP^PTB^ and MI^PTB^ specimens was described in beta diversity analysis.

**FIGURE 3:**
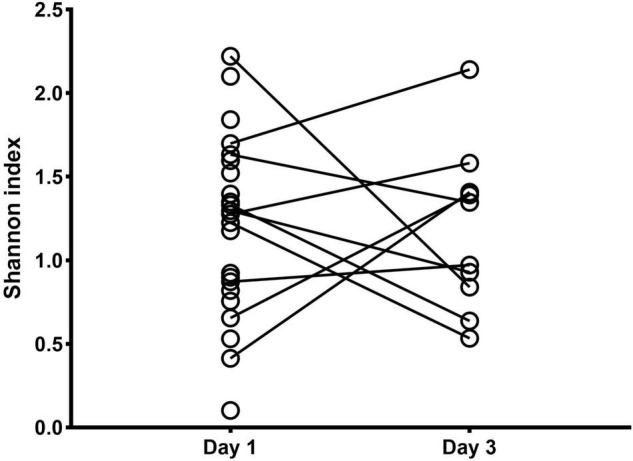
Alpha diversity analysis of the lung bacterial communities in newborns at days 1 and 3 after birth. Shannon index was measured on bronchoalveolar lavage samples at days 1 and 3 after intubation for MI^PTB^ and SP^PTB^ infants’ groups.

Ten on twenty-three newborns were still intubated on the third day after birth and a second BALF sample was collected (3 in the SP^PTB^ and 7 in the MI^PTB^ group, respectively). Alpha diversity as Shannon index was computed showing no differences between days 1 and 3. At day 3 after birth the average value of Shannon index was equal to 1.80 in the SP^PTB^ group vs. 1.61 MI^PTB^ in the group, *p* = 0.65) (Figure 3). Likewise, no differences were observed in terms of relative abundances of the detected species (data not shown) suggesting that no main changes occurred after birth at days 1–3.

The analysis was limited to the BALF samples of the first and third day of life due to the low number of BALF samples at day 7 (a total of five specimens: 3 in the SP^PTB^ and 2 in the MI^PTB^ group, respectively).

**FIGURE 4:**
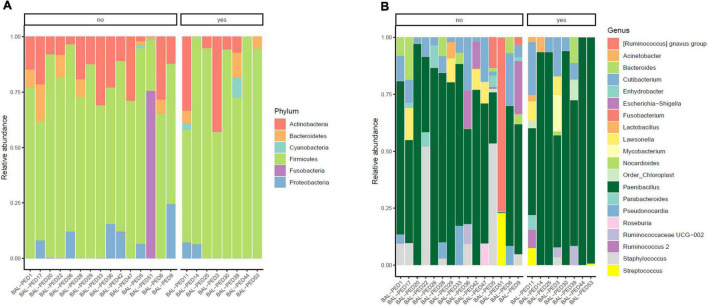
Relative abundances of bacterial taxa composing the lung bacterial communities in hospitalized newborns. For each sample of the BPD and noBPD groups, proportions for major phyla **(A)** and major genera **(B)** were computed, normalized, and presented as stacked bar plots. The statistical significance at a *P*-value of ≤ 0.05 was assessed using the Mann–Whitney *U*-test.

**FIGURE 5:**
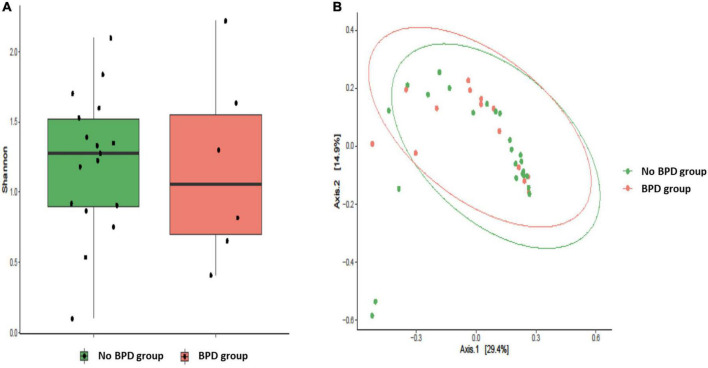
Alveolar microbiota Alpha and Beta diversity analysis of preterm newborns classified on the basis of Bronchopulmonary Dysplasia (BPD) diagnosis. Alpha diversity was evaluated by Shannon index measure. The values obtained were compared, resulting in no statistically significant difference between No BPD and BPD groups (Mann–Whitney *U*-test) **(A)**. Comparison for Beta diversity analysis was made using Jaccard distance. The principal coordinate analysis (PCoA) results are presented as two-dimensional ordination plots, which were generated using two principal coordinates (i.e., axis 1 and axis 2) **(B)**. The statistical significance at a *P*-value of ≤ 0.05 was assessed using the permutational multivariate analysis of variance (PERMANOVA).

In our cohort, eight out of 23 infants developed BPD, so that we investigated lung microbial communities in infants who developed BPD vs. those who did not develop BPD. By analyzing the BALF samples on day 1 of life, both BPD and No BPD groups showed a predominance of *Firmicutes* and *Actinobacteria* in addition to the presence of *Proteobacteria*, *Fusobacteria*, *Cyanobacteria*, and *Bacteroidetes* without a characteristic microbiological signature, linked to disease development (Figure 4A). Similarly, no differences at genus level were detected (Figure 4B). No significant differences were underlined by alpha diversity and beta diversity analysis (Figure 5).

Finally, we harvested meconium specimens for each infant to search for a microbial signature related to SP^PTB^ and MI^PTB^. Intriguingly, we were not able to detect any signal after 16S rDNA V3-V4 amplification.

### Vaginal Swabs

In order to identify a specific microbial signature in MI^PTB^ and SP^PTB^, we investigated the mothers’ vaginal microbiota. Twenty vaginal swabs were analyzed for vaginal microbiota composition (9 in the MI^PTB^ due to the presence of two pairs of twins and 11 in the SP^PTB^ due to the presence of a pair of twins). A total of 4,056,491 high-quality sequences comprising 7,145 unique ASVs were obtained. All 20 sequenced samples showed yielded ≥ 17,000 sequences with a mean of 202,824 and median of 224,678, range 17,214–349,108 reads/sample. After removing taxa with prevalence lower than 0.01%, a total of 3,590,745 reads were obtained 511 ASVs.

**FIGURE 6:**
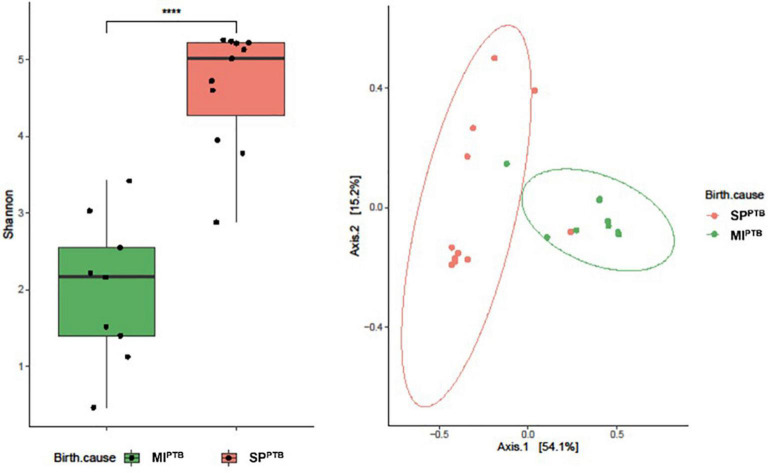
Alpha **(A)** and Beta **(B)** diversity analysis of the vaginal bacterial communities of the newborns’ mothers, classified on the basis of preterm birth subtype (MI^PTB^ and SP^PTB^). Alpha diversity was evaluated by using Shannon index measure, and the values for MI^PTB^ and SP^PTB^ infants were compared **(A)**. The statistical significance at a *P*-value of ≤ 0.05 (****) was assessed using the Mann–Whitney *U*-test. Beta diversity analysis was measured using Jaccard distance, computed for MI^PTB^ and SP^PTB^ groups. The principal coordinate analysis (PCoA) results are presented as two-dimensional ordination plots, which were generated using two principal coordinates (i.e., axis 1 and axis 2). The statistical significance at a *P*-value of ≤ 0.05 was assessed using the permutational multivariate analysis of variance (PERMANOVA).

We found that Shannon Diversity index was significantly higher in SP^PTB^ compared to MI^PTB^ (4.63 ± 0.78 vs. 1.98 ± 0.94, respectively, *p* < 0.05) suggesting that variation in bacterial population was associated with the two groups (Figure 6A). Particularly, SP^PTB^ group showed an increased Pielou’s evenness (0.79 ± 0.13) in comparison with the MI^PTB^ group (0.34 ± 0.15) that indicated an increasing prevalence of some bacterial species in the latter group (data not shown). To evaluate the similarity of microbiota profiles, Jaccard beta diversity was computed and plotted as PCoA graph. Interestingly, microbial communities appeared diversely distributed with a minimal overlap between SP^PTB^ (red) and MI^PTB^ (green) samples. Nonetheless, individual profiles of the two groups cluster on different spatial level as portrayed by ellipses. PERMANOVA statistic test confirmed our representation (*p* < 0.05) (Figure 6B).

**FIGURE 7:**
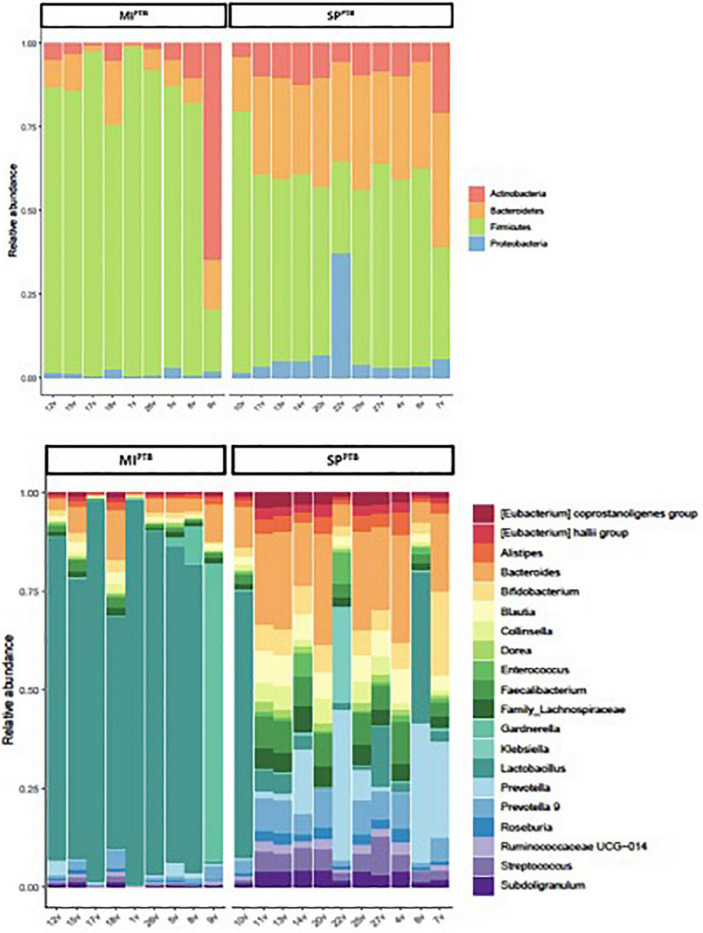
Relative abundances of bacterial taxa composing the newborns’ mothers vaginal flora, classified on the basis of preterm birth type (MI^PTB^ and SP^PTB^). For MI^PTB^ and SP^PTB^ groups, proportions for major phyla **(A)** and major genera **(B)** were computed, normalized, and presented as stacked bar plots, respectively. The statistical significance at a *P*-value of ≤ 0.05 was assessed using the Mann–Whitney *U*-test.

Both SP^PTB^ and MI^PTB^ groups showed a predominance of *Firmicutes*, *Proteobacteria*, *Actinobacteria*, and *Bacteroidetes*. However, there was a characteristic feature in the microbiota of women who have had a delivery for SP^PTB^ vs. the microbiota in women who have had a cesarean section for MI^PTB^. Indeed, SP^PTB^ group showed a significant decreasing in *Firmicutes* (54% vs. 79%, *p* = 0.0031) and the consequent increasing *Bacteroidetes* (31% vs. 9%, *p* = 0.00002). Intriguingly, a slight increasing in relative abundance of *Proteobacteria* (5.5% vs. 1.3%), rather than *Actinobacteria* (showing an average around 10% for both groups), was observed in MI^PTB^ group (Figure 7A).

As expected, Lactobacillus genus was decreased in SP^PTB^ group vaginal swabs in comparison with MI^PTB^ group (Figure 7B). Relative abundances of the other genera consequently showed an increasing.

### Cultural and Molecular Analyses Results

Among BALF samples collected on the first day of life, only 2 of them were positive for *Ureaplasma parvum*. *Ureaplasma parvum* was molecularly identified in only one BALF collected on the 7th day of life. All three newborns with positive BALF for *Ureaplasma parvum* developed BPD, with *Ureaplasma parvum* BALF positivity statistically correlating to the development of BPD (*p* = 0.03, data not shown in Table 2). The vaginal swabs of the mothers of *Ureaplasma parvum*-positive infants also showed positivity for the pathogen.

Culture of all other BALF specimens were negative, except for BALF on days 3 and 7 of a BPD infant who tested positive for *Staphylococcus haemolyticus*.

Concerning vaginal swab exams, in SP^PTB^ group 9 out of 12 (75%) vaginal swabs were positive (4 = *Ureaplasma parvum*, 1 = *Klebsiella pneumoniae*, 2 = *Candida albicans*, 1 = *Streptococcus agalactiae*, 1 = *Escherichia coli*), in MIPTB group 5 out of 11 (45%) vaginal swabs were positive (3 = *Ureaplasma parvum*, 1 = *Escherica coli*, 1 = *Citrobacter koseri*).

Considering BPD/No-BPD groups, 4 vaginal swabs in the BPD group (50%) and 3 in the No-BPD group (20%) were positive for *Ureaplasma parvum* (*p* = 0.18, data not shown in Table 2). In the BPD group a vaginal swab was positive for *Citrobacter koseri* and another one for *Klebsiella pneumoniae*, while 2 vaginal swabs were negative (25%). In the no-BPD group 7 vaginal swabs were negative (47%), while, in addition to the three *Ureaplasma parvum* positive vaginal swabs, 2 were positive for *Escherichia coli*, 1 for *Streptococcus agalactiae* and 2 for *Candida albicans*. The cultures of the meconium samples were all negative.

## Discussion

The present paper aims to identify specific vaginal and neonatal lung microbiota profiles associated to the occurrence of SP^PTB^ and MI^PTB^. As preterm births have been historically considered a sign of infection, we compared SP^PTB^ cases to MI^PTB^ cases, to understand if an adverse uterine environment could influence preterm birth and neonatal lung microbiota.

Chorioamnionitis, defined as inflammation of fetal membranes, is one of the mechanisms leading to preterm labor. A clinical diagnosis can be made prenatally by maternal findings (fever, uterine contractions, leucocytosis, high serum levels of C-reactive protein, abnormal vaginal secretions), while a pathological diagnosis is obtained postnatally by histological examination of the placenta and the amniochorionic membranes (27). A proven microorganism infection can be detected only in 50% of cases of chorioamnionitis, probably due to the technical limitations of cultural tests. Microbiota profiling by NGS technique might represent a more sensitive diagnostic tool to identify dysbiosis and increased risk of SP^PTB^ (28, 29).

Recent studies challenged the assumption of a sterile uterine environment, demonstrating the presence of bacteria in the placenta, fetal membranes, and amniotic fluid (2–4). However, these findings are controversial as there is the risk of contamination of low microbial biomass samples.

Vaginal dysbiosis is recognized as a factor that increases the risk of SP^PTB^. Several species of *Lactobacilli* promote the maintenance of an acid pH and the production of antimicrobial substances.

During pregnancy there is a progressive increase in abundance of species such as *Lactobacillus vaginalis, L. crispatus*, *L. gasseri*, and *L. jensenii*, belonging to the Phylum of the Firmicutes. Conversely, bacteria such as Prevotella, Sneathia, Gardnerella Vaginalis, Ruminococcaceae, Parvimonas, and Mobiluncus, often associated with bacterial vaginosis and widely present in non-pregnant women of childbearing age, progressively decrease (30). Hence, the microbiota of women in pregnancy becomes progressively more stable, less prone to changes and protective against genitourinary tract infections (31).

Several studies have shown that greater α-diversity of vaginal microbiota is associated with a progressive reduction in the relative abundance of the genus Lactobacillus, which is known for its role in giving stability to the vaginal microbiota and protecting it from pathogenic insults.

Consistently with previous literature, in this study we documented a greater alpha diversity of vaginal microbiota of the SP^PTB^ mothers when compared with mothers of the MI^PTB^ group. Moreover, in the SP^PTB^ group, vaginal swabs revealed a decrease in Firmicutes and an increase in abundance of Bacteroidetes. At Genus analysis, a significant decreased abundance of Lactobacillus was found in SP^PTB^.

The timeline of lung microbial colonization is highly debated. Mourani and colleagues reported the presence of detectable bacterial DNA in only 2 of 10 tracheal aspirates of intubated preterm infants in the first 72 h of life, whereas all samples from the same newborns were positive at day 7 (17). On the other hand, Lohmann et al. found the presence of bacterial DNA in all tracheal aspirates collected in 25 neonates immediately after orotracheal intubation on the first day of life (16). Lal et al. observed that BALF samples of extremely low birth weight infants exposed to chorioamnionitis showed a decreased genus Lactobacillus at birth, which has been associated with development of BPD.

All cases of chorioamnionitis were determined by positive placental histopathology and prenatal antibiotics administration in mothers (32).

We investigated any possible cause of spontaneous birth and possible adverse uterine environment (2 mothers in SP^PTB^ had chorioamnionitis and 10 had a p-PROM) looking for a microbial signature suggestive of spontaneous birth. However, despite differences observed in maternal vaginal swabs, we did not find in neonatal BALF samples at birth a different pattern of alpha and beta diversity and/or differences either at Phylum and Genus level, comparing SP^PTB^ and MI^PTB^ group.

A problem underlying the analysis of neonatal lung microbiota is the difficulty in retrieving material from the lungs and distal airways in neonatal population, due to the low biomass with bacterial loads close to the detection limit of the assays and to the high risk of contamination from the upper airways (33). In these terms, lung microbiota at first day of life could be significantly impaired by contamination during sampling and by sequencing technique. Moreover, samples derived from sites with a low microbial biomass can give results which are difficult to distinguish from DNA traces present in reagents used for extraction, amplification, and sequence library preparation for molecular microbiology studies (34, 35). Hence, we cannot exclude that our findings are the results of high sensitivity of sequencing process and sampling.

Indeed, the presence of bacterial DNA in BALF samples, while lending support to the hypothesis that the process of microbiota establishment might begin antenatally, does not exclude the possibility of post-birth microbiome development, impaired by environmental factors, or sample contaminations.

On the other hand, the lack of bacterial DNA in the analyzed meconium samples (collected before the start of enteral feeding, with no probiotic intake and in the absence of abdominal pathological processes) supports the idea that microbiota composition is acquired after birth, and it is not related with the cause of preterm birth or transferred from swallowing bacteria previously translocated from uterine niche in the amniotic liquid.

The absence of a specific microbial signature in the BALF samples of the two groups (MI^PTB^ vs. SP^PTB^) may, at least in part, suggest that the cause of preterm birth was not associated with infant lung microbiota composition.

While the two groups differed for some clinical characteristic as GA and birth weight, the analysis conducted on BALF samples of infants who developed or not BPD, did not show statistically significant differences.

Furthermore, the newborns of the BPD group had a higher incidence of hemodynamically significant PDA, blood stream infections and pneumonia during hospitalization and, consequently, are subjected to a greater number of antibiotic therapy cycles (Table 2). However, the two groups did not differ in the use of intrapartum antibiotic prophylaxis, nor in the time elapsed from birth to the start of antibiotic therapy. This may partly justify the lack of a statistically different expression of the microbiota in BALF samples taken in the first 24 h of life, and certainly more information could have been obtained by having a greater number of BALF samples available on the third, and seventh day and in the more advanced periods of life.

Although not statistically significant, a slight reduction in Shannon diversity index is evident in the BALF samples of BPD infants (Figure 5). This trend confirms the results obtained by Lohmann et al. (16). The authors identified a reduction in the number of species of respiratory microflora and Shannon diversity index in the BALF samples obtained in the first 24 h of life from 10 infants diagnosed with BPD compared to 15 other infants who had not developed the disease. A decrease in microecological diversity of the respiratory tract in BPD was observed also by Lal et al. (32) who analyzed 18 newborns with confirmed BPD, of which 5 infants with an extremely low birth weight.

We did not find a characteristic microbiological signature linked to BPD development both at genus and phylum level. However, the results of the studies by Lohmann et al. and Lal et al. showed opposite trends in Proteobacteria (a downward trend in the results of Lohmann et al. compared to an increase in Lal et al.) and Firmicutes abundances (with an upward trend in Lohmann et al. compared to a decrease in Lal et al.).

Collaterally, from cultural analysis, positive *Ureaplasma parvum* day-one BALF culture resulted significantly more prevalent in BPD group. Moreover, all neonates with positive day-one BALF were born form mother with positive vaginal swabs for Ureaplasma. This result is in line white previous studies, which associated Ureaplasma colonization in the respiratory tract with the subsequent development of BPD (16, 17, 36, 37).

However, there is still not a total agreement on the relationship between respiratory Ureaplasma colonization and BPD development, as well as there is not agreement on the benefit or risk of antibiotic therapy for respiratory Ureaplasma colonization (38).

We are aware that our study may have several limitations, as the small study population, given the difficulty of obtaining vaginal swabs timely before birth and the difficulty of sampling from the distal airways in the neonatal population. Other limitations of the study are represented by the lack of analysis of the placental microbiota and the lack of a histological diagnosis of chorioamnionitis. The implementation of these analysis, in association with further studies on amniotic fluid microbiota are needed to investigate the presence of a link between maternal and neonatal microbiota.

## Conclusion

An increase in α-diversity values and a consequent fall in Lactobacillus in vaginal environment could be associated to a higher risk of SP^PTB^, probably due to a less stable and protective environment, suggesting that variation in maternal microbiota can predispose to SP^PTB^.

We could identify neither a specific neonatal lung or meconium microbiota profiles in preterm infants born by SP^PTB^ nor a microbiota at birth suggestive of subsequent BPD development. However, accordingly to previous studies, the presence of Ureaplasma bacteria in newborns’ BALF could be considered a risk factor for the development of BPD.

Although a strict match has not been revealed between microbiota of SP^PTB^ mother-infant couples, a relationship cannot be excluded.

To figure out the reciprocal influence of the maternal-neonatal microbiota and its potential role in the pathogenesis of SP^PTB^ and BPD further research is needed.

## Data Availability Statement

The data presented in this study are deposited in the sequence read archive (SRA) and are available as BioProject number PRJNA849991.

## Ethics Statement

The studies involving human participants were reviewed and approved by Comitato Etico Fondazione Policlinico Universitario A. Gemelli IRCCS, Rome, Italy. Written informed consent to participate in this study was provided by the participants’ legal guardian/next of kin. Written informed consent was obtained from the individual(s), and minor(s)’ legal guardian/next of kin, for the publication of any potentially identifiable images or data included in this article.

## Author Contributions

CTi, AP, and FD conducted the statistical analysis and interpretations of results. CTi designed the study. AP, CTi, and FD wrote the manuscript with the support of CTe, SD’I, ND, ALa, GSc, and GV. FD, FM, GSa, DB, BP, and MS carried out the microbiological analysis. CTi, AP, CTe, SD’I, ND, MT, ALi, and NM collaborated in data collection. All authors read and approved the submitted version.

## Conflict of Interest

The authors declare that the research was conducted in the absence of any commercial or financial relationships that could be construed as a potential conflict of interest.

## Publisher’s Note

All claims expressed in this article are solely those of the authors and do not necessarily represent those of their affiliated organizations, or those of the publisher, the editors and the reviewers. Any product that may be evaluated in this article, or claim that may be made by its manufacturer, is not guaranteed or endorsed by the publisher.

## Footnotes

1
https://docs.qiime2.org/2021.8/plugins/available/feature-classifier/classify-sklearn/

2
https://www.arb-silva.de

3
https://www.rstudio.com/
